# Bullous Systemic Lupus Erythematosus: Clinical Presentation in Two Asian Cases

**DOI:** 10.1155/carm/1980187

**Published:** 2026-05-26

**Authors:** Phuong Thi Thanh Nguyen, Thuy Thi Phan Nguyen, Hoang Vu Nguyen, Anh Thi Minh Nguyen, Nhi Thi Uyen Pham

**Affiliations:** ^1^ Clinial Trial Unit, Ho Chi Minh City Hospital of Dermato-Venereology, Ho Chi Minh City, Vietnam; ^2^ Faculty of Medicine, University of Health Sciences, Viet Nam National University, Ho Chi Minh City, Vietnam; ^3^ Clinical Department, Ho Chi Minh City Hospital of Dermato-Venereology, Ho Chi Minh City, Vietnam

**Keywords:** bullous disease, bullous systemic lupus erythematosus, immunobullous disease, lupus erythematosus.

## Abstract

Bullous systemic lupus erythematosus (BSLE) is a rare subset of systemic lupus erythematosus (SLE), characterized by the rapid onset of vesiculobullous lesions that primarily affect sun‐exposed areas of the body. These lesions typically form along the dermal–epidermal junction and are associated with the presence of autoantibodies that target the skin. While BSLE often occurs in patients with a prior diagnosis of SLE, it can occasionally present as the initial manifestation of the disease. The diagnosis of BSLE requires a thorough evaluation based on clinical features, immunofluorescence studies, histological findings, and serological testing to distinguish it from other similar dermatological conditions. Herein, we report two cases of BSLE at a tertiary Dermatology Hospital in Vietnam.

## 1. Introduction

Bullous systemic lupus erythematosus (BSLE) is a rare, distinctive vesiculobullous phenotype within systemic lupus erythematosus (SLE) that can clinically resemble other subepidermal blistering diseases. Contemporary reviews emphasize its sudden onset of tense bullae on normal or erythematous skin, frequent involvement of sun‐exposed sites and/or mucosa, and typically nonscarring resolution, while also underscoring how easily it can be mistaken for bullous pemphigoid, linear IgA dermatosis, or epidermolysis bullosa acquisita (EBA) [1].

In keeping with current diagnostic frameworks, confirmation relies less on morphology alone and more on clinicopathologic correlation: a neutrophil‐rich subepidermal blister on histology, supportive immunofluorescence when available, and lupus‐specific serology/systemic features [2, 3]. At the same time, the literature highlights practical gaps, particularly limited access to IIF/ELISA for anti‐Type VII collagen antibodies and immunoelectron microscopy, meaning that real‐world diagnosis often depends on integrating the available pathology with the broader SLE context [1, 4–6].

We present two cases that mirror these contemporary themes and clarify key clinical takeaways: (i) BSLE as the initial clue to SLE in an older woman with predominantly facial, sun‐exposed bullae and a supportive direct immunofluorescence (DIF) pattern, and (ii) BSLE in a young man with lupus nephritis in whom DIF was negative after prior systemic therapy, illustrating that a negative DIF does not preclude BSLE when histology and lupus serology are concordant [1, 7, 8].

## 2. Case 1

A 50‐year‐old female patient presented to the HCMC Hospital of Dermato‐Venereology with multiple tense vesicles and bullae on her face. The eruption initially appeared as a pruritic vesicle on her right cheek 1 month prior, gradually spreading across her face. She sought medical attention 1 week later and was initially diagnosed with pemphigus vulgaris. Treatment was initiated with oral prednisolone at 0.5 mg/kg/day; however, there was no clinical improvement.

Her personal and family medical histories were unremarkable for any confirmed autoimmune or blistering disorders.

On examination, multiple tense vesicles and bullae, ranging in size from 0.2 to 1 cm, were observed on a normal‐appearing base. These vesiculobullous lesions were distributed across her cheeks, eyelids, nose, chin, upper cutaneous lip, and forehead. In addition, a 3‐mm erosion was noted on the oral mucosa.

Nikolsky’s sign was negative. No lymphadenopathy or signs of arthritis were detected, and the remainder of the physical examination was unremarkable.

Laboratory evaluation revealed positive anti‐SSA antibody and positive antiribosomal P antibody.

**FIGURE 1:**
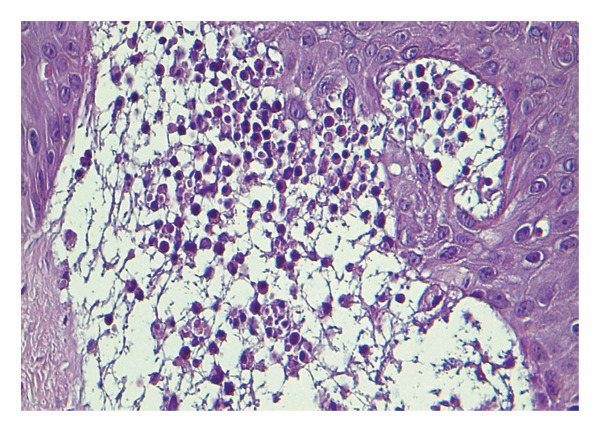
Case 1. Histopathologic findings revealed a subepidermal blister with neutrophils and inflammatory cells in the bulla.

Skin histopathology demonstrated subepidermal cleavage with a predominantly neutrophilic inflammatory infiltrate (Figure 1). DIF showed linear IgG and C3 deposition at the basement membrane zone (BMZ), while IgM and IgA were negative.

Based on clinical and laboratory findings, a diagnosis of BSLE was confirmed.

**FIGURE 2:**
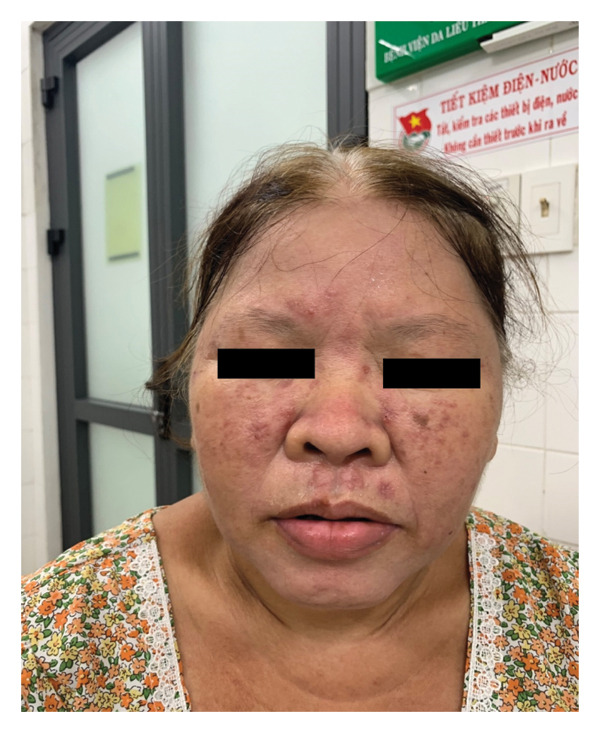
Case 1. The patient was followed up 1 week later with erythematous macules on her cheeks, forehead, nose, chin, and cutaneous upper lip. No new lesions appeared.

The patient was treated with oral prednisolone at 1 mg/kg/day in combination with hydroxychloroquine 200 mg/day. After 1 week of treatment, the skin lesions showed marked improvement (Figure 2).

The patient continued to be followed up every 2 weeks, with a gradual tapering of prednisolone. During the 2‐month treatment period, no new lesions developed.

## 3. Case 2

A 22‐year‐old male patient was hospitalized with slightly pruritic and stinging tense bullae on his chest, abdomen, and arms, which had developed over the past four days. The eruption initially appeared as a pruritic bulla on the chest and gradually spread to the abdomen and arms before presentation.

The patient reported a history of SLE complicated by hypertension and lupus nephritis, diagnosed by an internal medicine physician at a tertiary general hospital 12 days prior. He had been receiving treatment with methylprednisolone (0.75 mg/kg/day), mycophenolate (1 g/day), losartan (50 mg/day), calcium lactate pentahydrate (1 g/day), and folic acid (5 mg/day).

After 8 days of treatment, he developed fluid‐filled bullae, which rapidly spread across the chest, abdomen, and arms. Due to a lack of clinical improvement, he was admitted to the HCMC Hospital of Dermato‐Venereology 4 days later.

His family history was unremarkable for any confirmed autoimmune or blistering disorders.

On admission, the patient appeared fatigued, though vital signs were within normal limits.

**FIGURE 3:**
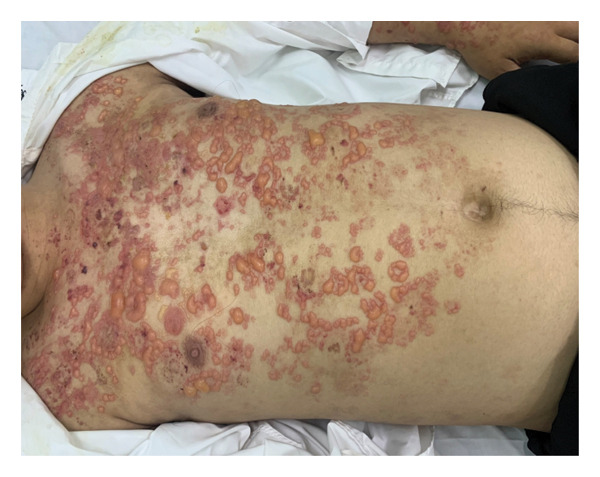
Case 2. Tense vesicles and bullae over normal‐appearing and erythematous skin on the patient’s chest, abdomen, and arms.

Skin examination revealed multiple tense vesicles and bullae measuring 0.5–1 cm in diameter, distributed over both normal and erythematous skin on the chest, abdomen, and arms (Figure 3). A 0.5‐cm ulcer was noted on the hard palate, and nonscarring diffuse hair loss was observed. Nikolsky’s sign was negative. There were no signs of nail fold telangiectasia, nail plate abnormalities, lymphadenopathy, or arthritis.

Laboratory evaluation showed positive anti‐Smith, anti‐RNP, anti‐Ro52, anti‐SSA, anti‐SSB, and antidouble‐stranded DNA (dsDNA) antibodies (titer ≥ 1:1000). Complement levels were decreased, with C3 at 63.82 mg/dL (reference range: 67–149 mg/dL) and C4 at 8.55 mg/dL (reference range: 10–38 mg/dL).

Additional laboratory tests revealed anemia, with a hemoglobin level of 9.5 g/dL (reference range: 12.3–15.3 g/dL). Urinalysis indicated proteinuria.

**FIGURE 4:**
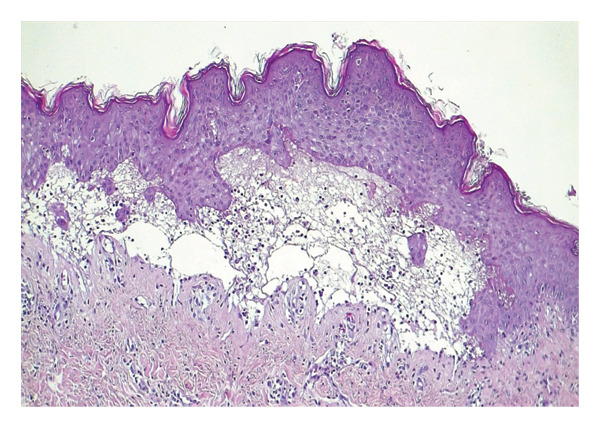
Case 2. Histologic feature showed subepidermal cleavage with neutrophil‐rich mixed inflammation infiltrate and mucin deposits.

Biopsy specimens were obtained, and histopathological examination revealed subepidermal cleavage, a neutrophil‐rich mixed inflammatory infiltrate, and mucin deposits in the reticular dermis (Figure 4). DIF was negative for IgG, IgA, IgM, and fibrinogen.

Based on these findings, a diagnosis of BSLE was confirmed. The patient was initiated on oral methylprednisolone (1 mg/kg/day) and hydroxychloroquine (200 mg/day), resulting in a gradual improvement of the dermatoses within two weeks.

**FIGURE 5:**
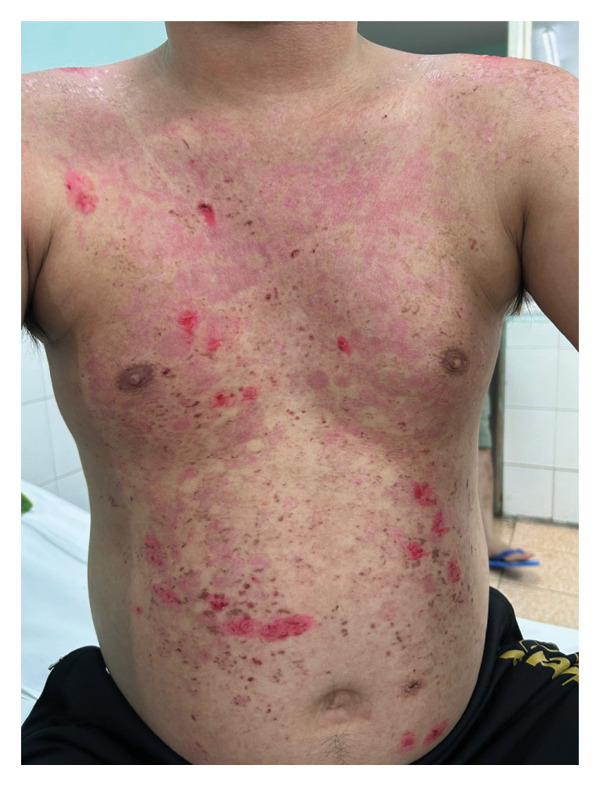
Case 2. The patient, after a 2 month follow‐up, showed significant improvement in the skin lesions (the pink color on his skin was due to the topical treatment solution).

Upon discharge, the patient continued treatment with prednisolone (1 mg/kg/day), hydroxychloroquine (200 mg/day), and an escalating dose of mycophenolate mofetil up to 500 mg/day. He was followed up every 2 weeks, with gradual tapering of prednisolone. No new skin lesions were observed during the 3‐month follow‐up period (Figure 5).

## 4. Discussion

BSLE is an uncommon but distinctive blistering manifestation within the spectrum of SLE, and current understanding is largely derived from case reports and small case series rather than large cohort studies [1, 7]. Rather than reiterating established pathogenic mechanisms, the present cases highlight several clinically relevant diagnostic and therapeutic challenges that are frequently encountered in contemporary practice.

A key diagnostic issue illustrated by our cases is the variable temporal relationship between BSLE and systemic disease. The first patient presented with BSLE as the initial clinical manifestation of SLE, a scenario that has been repeatedly described in both adult and pediatric populations [9–13]. In such settings, BSLE is particularly prone to misdiagnosis, as the abrupt onset of tense bullae may closely mimic pemphigus vulgaris, bullous pemphigoid, or other subepidermal blistering disorders. In contrast, the second patient developed BSLE in the context of recently diagnosed SLE with active lupus nephritis, underscoring that new‐onset blistering eruptions in patients with established systemic disease should prompt consideration of BSLE rather than being attributed solely to drug reactions or disease flares. This dichotomy reflects observations from larger reviews, which suggest that BSLE may either precede, coincide with, or follow the diagnosis of SLE, without a consistent relationship to overall disease duration [7].

Another important clinical insight from our series is the limited reliability of DIF as a sole diagnostic criterion. Although linear IgG deposition along the BMZ is reported in approximately 70%–80% of BSLE cases [14], negative DIF does not exclude the diagnosis. Our second case exemplifies this challenge, as DIF was negative despite characteristic histopathology and strongly supportive lupus‐specific serology. Similar observations have been reported in the literature, particularly in patients who have received prior systemic corticosteroids or immunosuppressive therapy, which may reduce immunoreactant deposition below the detection threshold of conventional DIF techniques [2, 8]. In addition, the absence of detectable circulating antibodies in Type VII collagen does not preclude BSLE Type I, as antibodies may be exclusively tissue‐bound and demonstrable only by immunoelectron microscopy, a modality that is not routinely available in many clinical settings [3, 15]. These findings reinforce the concept that BSLE remains a clinicopathologic diagnosis, requiring integration of clinical context, histology, and serologic features rather than reliance on immunopathology alone.

Histopathologically, both cases demonstrated subepidermal blistering with a neutrophil‐rich inflammatory infiltrate, a hallmark feature consistently emphasized in prior reports [1, 7]. The presence of dermal mucin in the second case further supported a lupus‐related blistering process and aided in differentiating BSLE from other neutrophilic subepidermal blistering disorders. This distinction is clinically relevant, as EBA typically shows greater skin fragility, healing with scarring and milia, and minimal dermal mucin, whereas bullous pemphigoid more often features eosinophil‐predominant inflammation and eosinophilic spongiosis [16].

Therapeutically, both patients demonstrated favorable responses to systemic corticosteroids combined with antimalarial therapy, consistent with prior reports indicating that BSLE often responds rapidly to lupus‐directed treatment [1, 11]. Dapsone is widely regarded as first‐line therapy for cutaneous control due to its efficacy against neutrophil‐mediated inflammation; however, systemic corticosteroids and additional immunosuppressive agents are frequently required when BSLE occurs in association with active systemic disease [1, 17]. Importantly, these cases suggest that treatment decisions should be driven by overall SLE severity and organ involvement rather than by immunopathologic subclassification of BSLE alone.

From a practical standpoint, the cutaneous prognosis of BSLE is often favorable with timely therapy, but relapses may occur and can coincide with systemic disease activity. Given the reported association between BSLE and lupus nephritis, follow‐up should include periodic assessment for systemic involvement, particularly urinalysis and renal function, in coordination with rheumatology or nephrology, alongside monitoring of serologic activity markers such as complement levels and anti‐dsDNA titers. Emerging and individualized approaches for refractory disease may include optimization of steroid‐sparing agents and, in selected cases, targeted or biologic therapies, with treatment choices guided by both skin control and overall SLE severity.

Due to the unavailability of indirect immunofluorescence and ELISA testing for anti‐Type VII collagen antibodies at our facility, a comprehensive clinical and pathological evaluation remains essential for accurate diagnosis and management.

In conclusion, these cases reinforce several clinically relevant messages supported by contemporary literature: BSLE should be considered across the full spectrum of SLE presentation, including as an initial manifestation; negative DIF does not exclude the diagnosis, particularly after prior therapy; and prompt recognition allows effective treatment using established lupus‐directed regimens without unnecessary escalation. An integrated clinicopathologic approach remains essential to achieving timely diagnosis and optimal management of BSLE.

## Funding

No funding was received for this study.

## Consent

Patient consent was obtained.

## Conflicts of Interest

The authors declare no conflicts of interest.

## Data Availability

The clinical data used to support the findings of this study are available from the corresponding author upon request.

## References

[1] Contestable J. J. , Edhegard K. D. , and Meyerle J. H. , Bullous Systemic Lupus Erythematosus: A Review and Update to Diagnosis and Treatment, American Journal of Clinical Dermatology. (2014) 15, no. 6, 517–524, 10.1007/s40257-014-0098-0, 2-s2.0-84911963887. 25358414

[2] Camisa C. and Sharma H. M. , Vesiculobullous Systemic Lupus Erythematosus: Report of Two Cases and a Review of the Literature, Journal of the American Academy of Dermatology. (1983) 9, no. 6, 924–933, 10.1016/s0190-9622(83)70210-0, 2-s2.0-0021083485. 6358284

[3] Camisa C. and Grimwood R. E. , Indirect Immunofluorescence in Vesiculobullous Eruption of Systemic Lupus Erythematosus, Journal of Investigative Dermatology. (1986) 86, no. 5, 10.1111/1523-1747.ep12355583, 2-s2.0-0022713268. 3528314

[4] Shirahama S. , Furukawa F. , Yagi H. , Tanaka T. , Hashimoto T. , and Takigawa M. , Bullous Systemic Lupus Erythematosus: Detection of Antibodies Against Noncollagenous Domain of Type VII Collagen, Journal of the American Academy of Dermatology. (1998) 38, no. 5, 844–848, 10.1016/s0190-9622(98)70472-4. 9591800

[5] Bain E. E. , Grover R. K. , Plunkett R. W. , and Beutner E. H. , Detection of Collagen VII Autoantibodies to NC1 and NC2 Domains of Collagen VII by ELISA in Suspected Epidermolysis Bullosa Acquisita and Bullous Lupus Erythematosus Patients, Journal of Dermatological Science. (2012) 65, no. 2, 155–156, 10.1016/j.jdermsci.2011.12.004, 2-s2.0-84856478809. 22225828

[6] Chan L. S. , Lapiere J. C. , Chen M. et al., Bullous Systemic Lupus Erythematosus with Autoantibodies Recognizing Multiple Skin Basement Membrane Components, Bullous Pemphigoid Antigen 1, laminin-5, laminin-6, and Type VII Collagen, Archives of Dermatology. (1999) 135, no. 5, 569–573, 10.1001/archderm.135.5.569, 2-s2.0-0032923729. 10328198

[7] de Risi-Pugliese T. et al., Clinical, Histological, Immunological Presentations and Outcomes of Bullous Systemic Lupus Erythematosus: 10 New Cases and a Literature Review of 118 Cases, Seminars in Arthritis and Rheumatism, 2018, Elsevier. 10.1016/j.semarthrit.2017.11.003 29191376

[8] Buschman K. E. , Seraly M. , Thong H. Y. , Deng J. , Draviam R. P. , and Abernethy J. L. , A Predominant IgG4 Subclass May be Responsible for False‐Negative Direct Immunofluorescence in Bullous Pemphigoid, Journal of Cutaneous Pathology. (2002) 29, no. 5, 282–286, 10.1034/j.1600-0560.2002.290504.x, 2-s2.0-0036020479. 12100628

[9] Barbosa W. S. , Rodarte C. M. , Guerra J. G. , Maciel V. G. , Fleury Júnior L. F. F. , and Costa M. B. , Bullous Systemic Lupus Erythematosus: Differential Diagnosis with Dermatitis Herpetiformis, Anais Brasileiros de Dermatologia. (2011) 86, no. 1, S92–S95, 10.1590/s0365-05962011000700024. 22068782

[10] Lourenço D. , Cunha Gomes R. , Aikawa N. E. , Campos L. M. A. , Romiti R. , and Silva C. A. , Childhood-Onset Bullous Systemic Lupus Erythematosus, Lupus. (2014) 23, no. 13, 1422–1425, 10.1177/0961203314544187, 2-s2.0-84909646700. 25074872

[11] Tincopa M. , Puttgen K. B. , Sule S. , Cohen B. A. , and Gerstenblith M. R. , Bullous Lupus: An Unusual Initial Presentation of Systemic Lupus Erythematosus in an Adolescent Girl, Pediatric Dermatology. (2010) 27, no. 4, 373–376, 10.1111/j.1525-1470.2010.01179.x, 2-s2.0-77954829021. 20653856

[12] Burke K. R. , Green B. P. , and Meyerle J. J. P. d. , Bullous Lupus in an 18-year-old, Pediatric Dermatology. (2011) 28, no. 4, 10.1111/j.1525-1470.2011.01502.x, 2-s2.0-79960752430. 21793895

[13] Fujimoto W. , Hamada T. , Yamada J. , Matsuura H. , and Iwatsuki K. , Bullous Systemic Lupus Erythematosus as an Initial Manifestation of SLE, The Journal of Dermatology. (2005) 32, no. 12, 1021–1027, 10.1111/j.1346-8138.2005.tb00894.x, 2-s2.0-32944462783. 16471470

[14] Vassileva S. , Bullous Systemic Lupus Erythematosus, Clinics in Dermatology. (2004) 22, no. 2, 129–138, 10.1016/j.clindermatol.2003.12.020, 2-s2.0-3042658256. 15234014

[15] Tani M. et al., Systemic Lupus Erythematosus With Vesiculobullous Lesions: Immunoelectron Microscopic Studies, Archives of Dermatology. (1984) 120, no. 11, 1497–1501, 10.1001/archderm.1984.01650470103024, 2-s2.0-84944965711. 6388508

[16] Kim J. and Kim S. C. , Epidermolysis Bullosa Acquisita, Journal of the European Academy of Dermatology and Venereology. (2013) 27, no. 10, 1204–1213, 10.1111/jdv.12096, 2-s2.0-84884902879. 23368767

[17] Hamminga E. A. and Vermeer M. H. , Bullous Systemic Lupus Erythematosus Responding to Mycophenolate Mofetil, European Journal of Dermatology. (2010) 20, no. 6, 844–845, 10.1684/ejd.2010.1081, 2-s2.0-78649880314. 20923751

